# Analysis of subconjunctival hemorrhage

**DOI:** 10.12669/pjms.291.2802

**Published:** 2013

**Authors:** Nedime Sahinoglu-Keskek, Selim Cevher, Ahmet Ergin

**Affiliations:** 1 Nedime Sahinoglu-Keskek, MD, Adana Numune Training and Research Hospital, Ophthalmology 01010 Yuregir, Adana, Turkey.; 2 Selim Cevher, MD, Adana Numune Training and Research Hospital, Ophthalmology 01010 Yuregir, Adana, Turkey.; 3 Ahmet Ergin, Professor, Adana Numune Training and Research Hospital, Ophthalmology 01010 Yuregir, Adana, Turkey.

**Keywords:** Etiology, Spontaneous, Subconjunctival hemorrhage, Trauma

## Abstract

***Objective:*** To determine associated conditions, gender distribution and location of subconjunctival hemorrhage (SCH).

***Methodology:*** This retrospective, observational and non-interventional study involved total of 50 patients with SCH aged 0.16-88 years. The conjunctiva was divided into 4 equal areas. The data about the subjects with SCH that includes age, gender, medical history, ocular history and location of hemorrhage were noted for all patients.

***Results:*** The patients with SCH consisted of 21 (42%) women and 29 (58%) men, with a mean age of 29.56 years. Of the 50 patients, 34 (68%) had traumatic and 16 (32%) had spontaneous SCH. Of traumatic SCH group 24 (70.6%) were men and 10 (29.4%) were women. SCH was more common in the temporal areas than other areas (40.5%).

***Conclusion:*** The most associated condition in spontaneous SCH was hypertension. SCH was found to be predominant in the temporal areas among all patients. In traumatic SCH, temporal areas were affected more, whereas in spontaneous SCH, nasal and temporal areas were affected equally. Traumatic etiology was more likely seen in men than women.

## Introduction

 Subconjunctival hemorrhage (SCH) is a common clinical condition of eye that is characterized by blood accumulation in the subconjunctival space. A ruptured vessel causes bleeding in the space between Tenon’s capsule and the conjunctiva. Generally it is a benign disorder and can be caused by trauma, hypertension, anticoagulant therapy, elevated venous pressure (Valsalva maneuver, coughing, vomiting) and acute hemorrhagic conjunctivitis.^1^^-^^8^ Elderly patients and patients with vascular disorders such as hypertension, arteriosclerosis and diabetes tend to have weak-walled conjunctival vessels that may crack easily under stress.^1^^,^^5^

 The objective of the study was to determine associated conditions, gender distribution and the incidence of SCH at each conjunctival location.

## Methodology

This was a retrospective study. Institutional Review Board approval was obtained.

***Inclusion Criteria:*** Consecutive patients with SCH attending our outpatient clinic were enrolled between January 2012 and June 2012. 

***Exclusion Criteria:*** Patients with other common causes of red eye such as conjunctivitis; episcleritis and scleritis; keratitis and corneal ulcer; iritis; glaucoma and other common conditions such as dry eye and blepharitis were excluded. Subconjunctival hemorrhages associated with globe rupture were also excluded. 

**Table-I:** Characteristics of the patients with subconjunctival hemorrhage.

*Variables*	*Spontaneous SCH*	*Traumatic SCH*	*Total*	*P-Value*
Number of cases	16	34	50	
Frequency	0.32	0.68	1	
Age				0.174
Mean	50.37	19.76	29.56	
SD	20.00	20.8	24.5	
Median	55	12	24	
Range	4-88	2 months-75		
Gender				0.009
Male	5	24	29	
Female	11	10	21	
Eye involvement				1.00
Right eye	8	17	25	
Left eye	8	17	25	
Entire conjunctiva				NA
Inferior	8	10	18	
Nasal	9	12	21	
Superior	4	4	8	
Temporal	9	23	32	
Total	30	49	79	

 Verbal informed consent was obtained from all study patients. Each patient’s age, gender, medical history and ocular history were assessed at the initial visit. The diagnosis of SCH was based on inspection and slitlamp examination. The medical history included the presence of systemic diseases, such as diabetes, hypertension, cardiovascular abnormality or any bleeding disorder, medications (e.g., aspirin, coumadin), eye rubbing, sneezing, heavy lifting, trauma and Valsalva. Clinical examination has included complete ocular examination including inspection, slitlamp examination and funduscopy. Patients with SCH were classified in two groups; traumatic and spontaneous. Traumatic SCH was defined as SCH resulting from trauma. Spontaneous SCH was defined as any SCH not related to trauma. Patients with spontaneous SCH were referred to general physicians to investigate existing systemic diseases and data from their clinical records were noted.

**Table-II:** Associated condition in spontaneous subconjunctival hemorrhage.

*Associated condition in spontaneous SCH*	*Number of patients (%)*
Not apparent	8 (50)
History of hypertension	4 (25)
Sneezing	3 (18.75)
Vomiting	1 (6.25)
Total	16 (100)

**Table-III:** Antihypertensive drugs used by hypertensive patients

*Patients*	*Drugs*
1	Olmesartan (Angiotensin II receptor blocker)
2	Olmesartan
3	Amlodipine (Calcium channel blocker)
4	Metoprolol (Selective beta 1 receptor blocker)

***Location of SCH: ***The anterior segment was examined with a slit lamp. Detailed drawings were used to record the results of slit lamp examination. The conjunctiva was divided into 4 equal areas as follows; superior(S), nasal(N), inferior(I), temporal(T). The location of the SCH was noted for each patient. If the SCH extended more than one area, each area was noted separately.

***Statistical Analysis: ***The unpaired Student’s test was used to compare mean values. For comparisons between the two groups, we applied the Chi2 test of independence test. A p value of ≤ 0.05 was considered significant.

## Results

 Fifty eyes of 50 patients with SCH were evaluated. They consisted 21 (42%) women and 29 (58%) men. Mean age was 29.56 (±24.92) with an age range 0.16-88 years. Table-I shows the characteristics of patients.

 Of the 50 patients with SCH, 34 (68%) had traumatic and 16 (32%) had spontaneous SCH. The majority of the spontaneous SCH group was female (68.8%) whereas it was male in traumatic group (70.6%). Men were more likely to have traumatic SCH (p=0.009). Forty-seven (94%) of patients with SCH were first episode and three (6%) were recurrence.

 Among the 16 patients with spontaneous SCH, hypertension was the common associated condition in four (25%) patients. (Table-II) These patients were using antihypertensive drugs. (Table-III) Patients with traumatic SCH were younger than patients with spontaneous SCH. Nature of trauma was blunt. Associated ocular findings were; periorbital ecchymosis in four patients, periorbital edema in three patients, intracameral hemorrhage in one patient and laceration on upper eye lid in one patient. The mean ages for traumatic and spontaneous SCH respectively were; 19.76 and 50.37.

 SCH was more common in temporal areas than other areas (40.5%). Table-IV shows the distribution of SCH. In 31 patients (62%) SCH was seen in only one area. Table-V shows extent of hemorrhage.

 The right and left eye were involved equally. There were no statistically significant differences between spontaneous and traumatic SCH with respect to eye involvement (p=1.00) and location of SCH (P=0.50).

**Table-IV:** Frequency of areas of subconjunctival hemorrhage

	*Frequency*	*Percent*
Superior	8	10.1
Nasal	21	26.6
Inferior	18	22.8
Temporal	32	40.5
Total	79	100

**Table-V:** Number of areas involved in all patients.

*Number of areas involved*	*Number of patients*	*%*
1	31	62
2	12	24
3	4	8
4	3	6
Total	50	100

## Discussion

 Although SCH is a common condition in ophthalmology there are only few studies about this. In this study we observed the associated conditions, gender distribution of patients with SCH and most seen conjunctival areas of the disease.

 Unlike previous studies, in this study the frequency of trauma in patients with SCH was higher up to 68%.^5^^,^^9^ In a study Mimura et al reported the ratio of the traumatic SCH as 8.7%.^5^ In another study Kaimbo et al found this ratio as 51.7%.^9^ We thought that high frequency of traumatic SCH is due to low socio economical level of the population. Also the region is an industrial and agricultural area. Work injuries are used to be seen in many clinics of the hospital.

Spontaneous SCH was most frequently associated with hypertension. This finding was consistent with previous studies.^2^^,^^9^ Other associated conditions were rare and included vomiting and sneezing.

 In our study the patients with SCH were young. The main reason for this was assumed to be that trauma was more common etiology in our patients. Also SCH was found more frequently in males in traumatic cases and females in spontaneous cases. The higher risk in male is probably related to working in heavy work and having more aggressive nature.

 SCH was more often found in temporal areas. In the traumatic patients with SCH it is an expected finding. There may be two reasons. One of them is protective effect of the nose for the nasal area. The other is large temporal bulbar conjunctiva. SCH is reported to be related to some other etiologies as; febrile systemic infections^10^^-^^13^ malaria^14^, carotid-cavernous fistula^15^ and delivery.^16^^,^^17^

## Conclusions

 In summary, we have presented an observational study about etiology, gender distribution and location of SCH. We found that traumatic SCH was seen more than spontaneous SCH in our region and young men are under risk of SCH. Spontaneous SCH is a cautionary clinical condition for systemic diseases such as diabetes, hypertension, cardiovascular and bleeding abnormalities. The patients with SCH must be referred to an internal medicine clinic for detailed systemic evaluation.
